# Sorption Profile of Low Specific Activity ^99^Mo on Nanoceria-Based Sorbents for the Development of ^99m^Tc Generators: Kinetics, Equilibrium, and Thermodynamic Studies

**DOI:** 10.3390/nano12091587

**Published:** 2022-05-07

**Authors:** Mohamed F. Nawar, Alaa F. El-Daoushy, Metwally Madkour, Andreas Türler

**Affiliations:** 1 Department of Chemistry, Biochemistry and Pharmaceutical Sciences, Faculty of Science, University of Bern, Freiestrasse 3, CH-3012 Bern, Switzerland; andreas.tuerler@unibe.ch; 2 Radioactive Isotopes and Generators Department, Hot Laboratories Center, Egyptian Atomic Energy Authority, Cairo 13759, Egypt; alaafeldaoushy@yahoo.com; 3 Chemistry Department, Faculty of Science, Kuwait University, Safat 13060, Kuwait; metwally.madkour@ku.edu.kw

**Keywords:** LSA ^99^Mo, CeO_2_ NPs, thermodynamic parameters, sorption kinetics, hydrothermal modification

## Abstract

^99^Mo/^99m^Tc generators play a significant role in supplying ^99m^Tc for diagnostic interventions in nuclear medicine. However, the applicability of using low specific activity (LSA) ^99^Mo asks for sorbents with high sorption capacity. Herein, this study aims to evaluate the sorption behavior of LSA ^99^Mo towards several CeO_2_ nano-sorbents developed in our laboratory. These nanomaterials were prepared by wet chemical precipitation (CP) and hydrothermal (HT) approaches. Then, they were characterized using XRD, BET, FE-SEM, and zeta potential measurements. Additionally, we evaluated the sorption profile of carrier-added (CA) ^99^Mo onto each material under different experimental parameters. These parameters include pH, initial concentration of molybdate solution, contact time, and temperature. Furthermore, the maximum sorption capacities were evaluated. The results reveal that out of the synthesized CeO_2_ nanoparticles (NPs) materials, the sorption capacity of HT-1 and CP-2 reach 192 ± 10 and 184 ± 12 mg Mo·g^–1^, respectively. For both materials, the sorption kinetics and isotherm data agree with the Elovich and Freundlich models, respectively. Moreover, the diffusion study demonstrates that the sorption processes can be described by pore diffusion (for HT-synthesis route 1) and film diffusion (for CP-synthesis route 2). Furthermore, the thermodynamic parameters indicate that the Mo sorption onto both materials is a spontaneous and endothermic process. Consequently, it appears that HT-1 and CP-2 have favorable sorption profiles and high sorption capacities for CA-^99^Mo. Therefore, they are potential candidates for producing a ^99^Mo/^99m^Tc radionuclide generator by using LSA ^99^Mo.

## 1. Introduction

There is an increasing interest in using ^99m^Tc (T_1/2_ = 6.01 h) for diagnostic purposes in nuclear medicine and radiotracing applications in the industry [1,2,3,4]. This increased interest has heightened the need to produce ^99^Mo/^99m^Tc generators on a large scale. The production of these generators includes different technologies, for instance, sublimation, electrochemical, solvent extraction, supported liquid membrane (SLM), and column chromatographic approaches [5,6,7,8,9]. Among these generators, the portable column chromatographic type is considered the primary source to supply ready-to-use ^99m^Tc onsite [7,10]. The idea of these generators is to retain the parent ^99^Mo on a sorbent for decay so that the daughter ^99m^Tc can then be easily separated in high purity by using an isotonic saline solution at desired time intervals [11,12]. 

The ^99m^Tc parent, ^99^Mo, can be produced in nuclear reactors either by fission of ^235^U or by direct neutron irradiation of natural Mo or enriched ^98^Mo targets [10,13]. On the one hand, fission produces more than 95% of the ^99m^Tc generators on the market because it provides ^99^Mo with high specific activity (>10^4^ Ci/g Mo). On the other hand, this route has some disadvantages. The main ones are the prohibitive production costs, the generation of large amounts of radioactive waste, and the increasing proliferation risks [14,15,16]. To overcome these difficulties, many methods have been suggested for using neutron-activated ^99^Mo of low specific activity (LSA) (~5–10 Ci/g Mo) [15,17,18,19,20]. However, the low sorption capacity of commercial materials is the most critical limitation, which reduces their applicability. It is worth mentioning that the retention capacity of alumina (Al_2_O_3_), the material used, is limited to only 2–20 mg.g^–1^ for molybdate (^99^MoO_4_^2−^) [8].

Recently, nanotechnology has shown that it has the potential to develop the properties of different materials. These newly developed materials provide attractive solutions in multidisciplinary sectors, such as biology, chemistry, and medical sciences [21]. Nanostructured materials possess a unique surface morphology, which derives from their small particle size structure, high surface area, and the large number of active sites. The successful synthesis of such nanostructured sorbents has been reported by using several approaches. Out of these techniques, wet-chemical precipitation and hydrothermal methods bring potential advantages. Wet chemical precipitation is a simple, cost-effective method that can be conducted at ambient temperature [22,23,24]. The hydrothermal route offers high product purity with uniform composition, less particle agglomeration, controlled surface morphology, and narrow particle size distribution [25,26]. Consequently, this unique surface morphology significantly improves sorption efficiency and high adsorbate selectivity [27]. In this context, the use of nanomaterial-based sorbents appears to be a promising solution to overcome the low sorption capacity of conventional sorbents for LSA ^99^Mo. These new sorbents might make it possible to produce ^99^Mo/^99m^Tc generators by using LSA ^99^Mo.

In this paper, our main objective is to evaluate the sorption profile of LSA ^99^Mo on several CeO_2_ nanosorbents developed in our laboratory. To achieve this goal, we report the preparation of CeO_2_ NPs using wet chemical precipitation and hydrothermal methods. Moreover, we investigated the sorption efficiency of the prepared materials for Mo under different experimental conditions such as pH, the initial concentration of the molybdate solution, contact time, and temperature. In addition, the sorption kinetics, equilibrium isotherm, and thermodynamics were evaluated.

## 2. Materials and Methods

### 2.1. Materials

All chemicals were of analytical grade purity and were used without further purification. Milli-Q water was used for the preparation of solutions and washings. Cerium nitrate hexahydrate Ce(NO_3_)_3_·6H_2_O, sodium hydroxide, ethanol absolute, and nitric acid were purchased from Merck, Darmstadt, Germany.

^99^Mo radiotracer solution was obtained by eluting 57.1 GBq fission ^99^Mo alumina-based ^99^Mo/^99m^Tc generator (Pertector, National Centre for Nuclear Research, Otwock, Poland) with 5 mL 1 M NaOH solution after ~7 d from the calibration date. The total ^99^Mo radioactivity was measured with a Capintec Radioisotopes Calibrator (model CRC-55tR Mirion Technologies, Inc., Florham Park, NJ, USA). The ^99^Mo eluate solution was passed through 0.45 micro-Millipore filter to retain any alumina particles. Then, the ^99^Mo solution was diluted with HNO_3_ solution to the desired pH value.

### 2.2. Instrumentation

Radiometric identifications and measurements were carried out by using a multichannel analyzer (MCA), Inspector 2000 model, Canberra Series, Mirion Technologies, Inc., Meriden, CT, USA, coupled with a high-purity germanium detector (HPGe). Samples of constant geometry were counted at a low dead time (<5%). The radioactivity levels were determined by quantifying the 740 keV photo peaks corresponding to ^99^Mo. A pH-meter with a microprocessor (Mettler Toledo, Seven Compact S210 model, Greifensee, Switzerland) was used to measure the pH values. A thermostated shaking water bath (Julabo GmbH, Seelbach, Germany) was used for all batch equilibrium studies. The X-ray powder diffraction (XRD) patterns were performed via a D8 ADVANCE diffractometer (Bruker AXS Inc, Madison, WI, USA) with a Cu-Kα radiation source (λ = 0.1542 nm) operating at 40 kV, 40 mA, and a scanning range of 2θ = 10–80° at room temperature. The Brunauer–Emmett–Teller (BET) surface area of the synthesized materials was measured using nitrogen sorption isotherms at −195 °C on a model Gemini VII, ASAP 2020 (Micromeritics Instrument, Norcross, GA, USA). Before the analysis, the sample was degassed for 12 h at 110 °C. Zeta potential (ζ) measurements were studied using a Zeta-sizer (Nano ZS, Malvern, UK) for isoelectric point (pH_IEP_) measurements. A JSM-7100F (JEOL) was used to conduct field emission scanning electron microscopy (FE-SEM) measurements.

### 2.3. Synthesis of Cerium Oxides Nanoparticles

#### 2.3.1. Wet Chemical Precipitation Method 

CeO_2_ NPs were prepared by a wet chemical method via the precipitation of cerium nitrate in an alkaline medium. In a typical procedure, 2 g of Ce(NO_3_)_3_·6H_2_O were dissolved in a mixture of Milli-Q water and ethanol absolute with a ratio of (1:4 *v*/*v*). Then, this solution was added drop-wise to a freshly prepared 0.4 M sodium hydroxide solution (pH~13) under continuous stirring, and the pH value was controlled at about 11. The mixture was stirred until a yellow suspension of CeO_2_ NPs was formed after approximately 30 min. After that, the formed precipitate was washed several times with de-ionized water and ethanol absolute so that the soluble salts and fine impurities could be removed. Subsequently, the washed precipitate was centrifuged at 4000 rpm for 15 min and dried in a drying oven at about 50–70 °C for 12 h. The resulting sample is denoted as CP (Chemical Precipitation).

#### 2.3.2. Hydrothermal Modification Method 

Hydrothermally modified CeO_2_ NPs were synthesized according to the same procedure as described in Section 2.3.1 with some modifications using a mixture of absolute ethanol and Milli-Q water (4:1 *v*/*v*). First, the formed NPs were transferred into a Teflon-lined stainless steel autoclave. Then, the autoclave was sealed and maintained at 150 °C for 12 h. Subsequently, the formed precipitate was washed several times with de-ionized water and ethanol absolute, centrifuged, and dried at about 70 °C for 12 h. The dried material is called HT (Hydrothermal Treatment).

**Figure 1:**
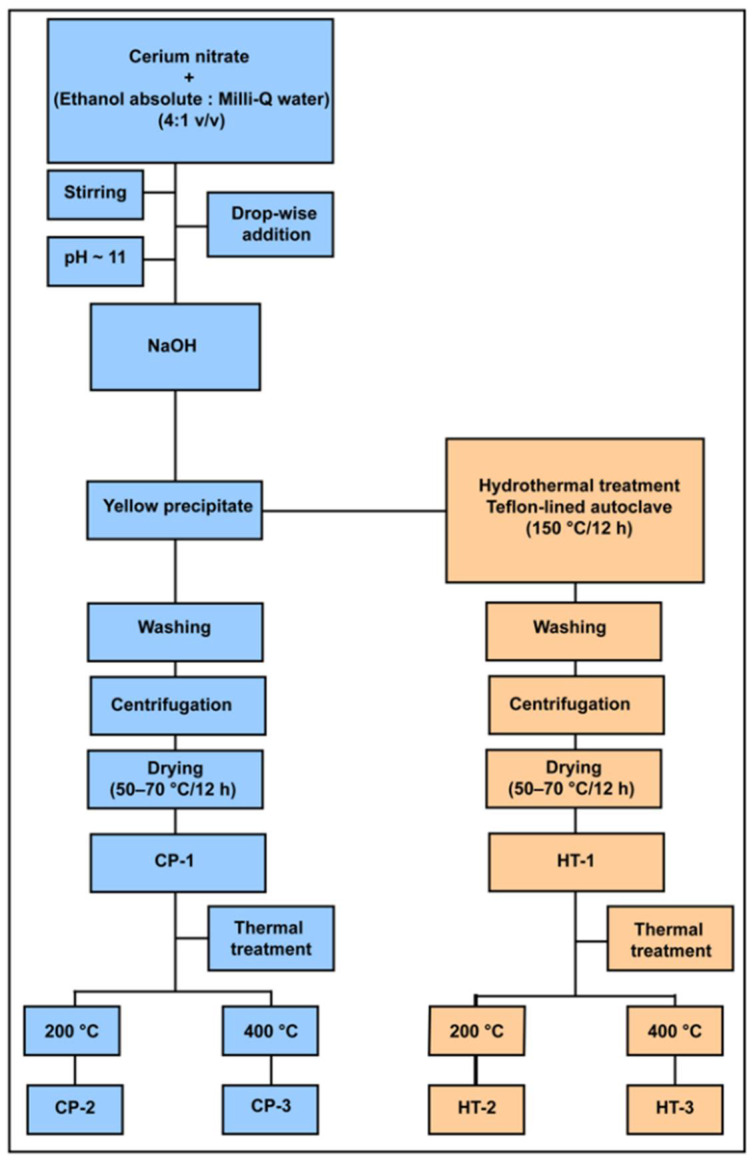
The synthesis protocol of CeO_2_ NPs using wet chemical precipitation and hydrothermal modification approaches.

Furthermore, the synthesized CeO_2_-CP and CeO_2_-HT NPs were heated at different temperatures: 200 °C and 400 °C with a heating rate of 10 °C/min for 2 h (Figure 1).

### 2.4. Batch Distribution Studies

#### 2.4.1. Effect of Solution pH

The influence of medium pH on carrier-added ^99^Mo uptake on each sorbent was evaluated at a wide range of pH values. For this purpose, we added 30 mL of molybdate solution (50 mg·L^−1^) to 300 mg of each sorbent. The uptake behavior was investigated at different pH values, which ranged from 1 to 12. The initial pH of the ^99^Mo solution was adjusted by using either HNO_3_ or NaOH. The mixtures were shaken in a thermostatic shaker at 25 ± 1 °C for 24 h. After that, the liquid phases were separated, centrifuged, and then 1 mL aliquot was withdrawn for radiometric measurements. In these analyses, we measured the total solutions radioactivity before and after equilibration with an HPGe counter by using an appropriate gamma-ray peak (740 keV). Additionally, both initial and equilibrium pH values were measured. Finally, the uptake percent of each material was calculated as a function of solution pH by using the following equation:

(1) $$\mathrm{Uptake}\%=\frac{A_{o}-{\;A}_{e}}{A_{o}}\times 100$$

where A_o_ and A_e_ are the initial and equilibrium of ^99^Mo radioactivity expressed in counts/min.

#### 2.4.2. Sorption Kinetics 

To estimate the sorption rate of carrier-added ^99^Mo on the prepared CeO_2_ NPs, we determined the uptake percent of ^99^MoO_4_^2−^ ions (~pH 3) at different time intervals. Typically, 300 mg of each sorbent were suspended in 30 mL of ^99^MoO_4_^2−^ solution (50 mg·L^−1^ in 0.001 M HNO_3_) and shaken in thermostated shaker bath at 25 ± 1 °C for different time intervals (5 min–50 h). Then, 1 mL of the supernatant was separated and measured. The sorption of Mo on each material was followed with time until the equilibrium was established. Finally, we calculated the Mo capacity ($Q_{t}$) in mg/g at each time (t) by using the following equation:

(2) $$Q_{t}=\frac{A_{o}-{\;A}_{t}}{A_{o}}\times C_{o}\times \frac{V}{m}$$

where A_o_ (count/min) is the initial activity, and $A_{t}$ is the ^99^Mo activity at a time (t), $C_{o}$ (mg·L^−1^) is the initial ^99^MoO_4_^2–^ ion concentration, V (L) is the total volume, and m (g) is the sorbent weight.

Furthermore, for a comprehensive understanding of the reaction kinetics and diffusion mechanisms, we used the obtained results and applied different sorption kinetic and diffusion models. 

#### 2.4.3. Equilibrium Sorption Isotherms

Sorption isotherm studies were conducted by varying the initial ^99^MoO_4_^2–^ ion concentration from 50 to 5000 mg·L^−1^ while keeping the sorbent dose constant. Herein, we mixed 30 mL of ^99^Mo solution (pH ~ 3) with 300 mg of each material. The other parameters, such as equilibrium time and temperature, were maintained at 24 h and 25 ± 1 °C, respectively. Subsequently, the aqueous phases were pipetted, centrifuged, and measured. The equilibrium adsorption capacity (Q_e_) in mg·g^−1^ was calculated using Equation (2). The obtained results were evaluated using sorption isotherm models.

In order to evaluate sorption isotherm models, three error distribution functions were used. These functions are Chi-square (X^2^), average percentage error (APE), and root mean square error (RMSE). These functions can be calculated according to the following equations:

(3) $$X^{2}=\sum \frac{{\left(Q_{e\left(\exp\right)}-{\;Q}_{e\left(\mathrm{cal}\right)}\right)}^{2}\;}{Q_{e\left(\mathrm{cal}\right)}}\;$$

(4) $$\mathrm{APE}=\left(\overset{N}{\underset{1}{\sum }}\left(\left|Q_{e\left(\exp\right)}-{\;Q}_{e\left(\mathrm{cal}\right)}\right|/Q_{e\left(\exp\right)}\right)/N\right)\times 100\;\;$$

(5) $$\mathrm{RMSE}=\sqrt{\left(1/N-2\right)\times \overset{N}{\underset{1}{\sum }}{\left(Q_{e\left(\exp\right)}-{\;Q}_{e\left(\mathrm{cal}\right)}\right)}^{2\;}}$$

#### 2.4.4. Temperature Effects

The change in molybdate uptake was studied under different reaction temperatures. The study was conducted between 298 and 343 K, and the other reaction conditions were kept constant. Moreover, to clarify the thermodynamic nature of the sorption process, we used the obtained data to investigate different thermodynamic parameters. These parameters are the standard enthalpy change (ΔH°), the standard entropy change (ΔS°), and the Gibbs free energy change (ΔG°).

## 3. Results and Discussion

In order to evaluate the sorption profile of the molybdate on the synthesized CeO_2_ NPs, different parameters were studied using the batch contact method. These parameters include solution pH, contact time, initial ion concentration (m_i_), and temperature. The impact of each parameter on the Mo sorption was studied separately. Meanwhile, the other factors were kept constant. 

### 3.1. Structural Characterization of the Prepared CeO_2_ NPs

**Figure 2:**
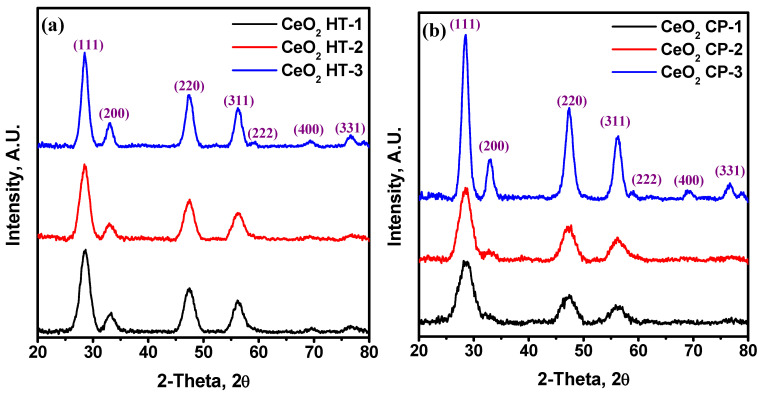
XRD patterns of CeO_2_ NPs at different temperatures prepared via (**a**) wet chemical precipitation method; (**b**) hydrothermal modification.

The XRD patterns of CeO_2_ nanoparticles prepared via wet chemical precipitation and hydrothermal methods are shown in Figure 2. The peaks are indexed using JCPDS card no: 34-0394. All samples of CeO_2_ nanoparticles have face centered cubic structure with the lattice parameters a = b = c = 5.411 Å and α = β = γ = 90°. The diffraction peaks were found at 28.56°, 33.08°, 47.47°, 56.36°, 59.08°, 69.40°, and 76.70° [28]. The absence of impurity indicates that both methods synthesize pure CeO_2_. The average crystallite size (D) of CeO_2_ nanoparticles is calculated by using the Debye–Scherrer equation:

(6) $$D=\frac{K\lambda }{\beta \cos\theta }$$

where λ is the wavelength of the Cu-Kα radiation, K is a constant (=0.89), θ is the diffraction angle, and β is the full width at half maximum (FWHM). The average crystallite size values for CeO_2_ nanoparticles were evaluated and listed in Table 1. The results revealed that the thermal treatment affects the crystallite size of the samples prepared by the wet chemical precipitation method. For these samples, elevating the temperature increases the crystallite size. However, in the case of the hydrothermal method, the thermal treatment does not influence the crystallite size.

**Figure 3:**
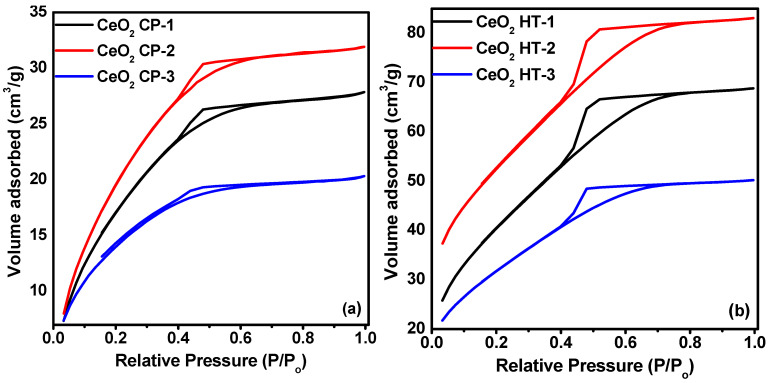
N_2_ adsorption–desorption isotherms of CeO_2_ NPs at different temperatures prepared via (**a**) wet chemical precipitation method; (**b**) hydrothermal modification.

We further examined the textural properties of the CeO_2_ nanoparticles using a nitrogen adsorption isotherm (Figure 3). All the samples exhibited Type IV isotherms with well-defined H_2_-type hysteresis loops, which indicates the mesoporous nature of the materials [29]. The BET surface area, pore volume, and pore size of all samples are tabulated in Table 1. The highest BET surface area of the mesoporous CeO_2_ is 187.2 m^2^·g^−1^. The surface area of the wet chemical precipitation method was increased upon increasing the temperature up to 200 °C and reached 175.3 m^2^·g^−1^, and further thermal treatment resulted in its reduction to 97.4 m^2^·g^−1^. Compared to CeO_2_ NPs prepared with the wet chemical precipitation method, the hydrothermally modified samples exhibit a higher surface area due to improved surface modification.

The zeta potential (ζ) measurements listed in Table 1 give insight into the surface charge. It revealed that the pH_IEP_ of CeO_2_ NPs is at about seven. Therefore, the surface of CeO_2_ NPs bears a positive charge below pH ~ 7 and a negative charge above this pH value.

**Figure 4:**
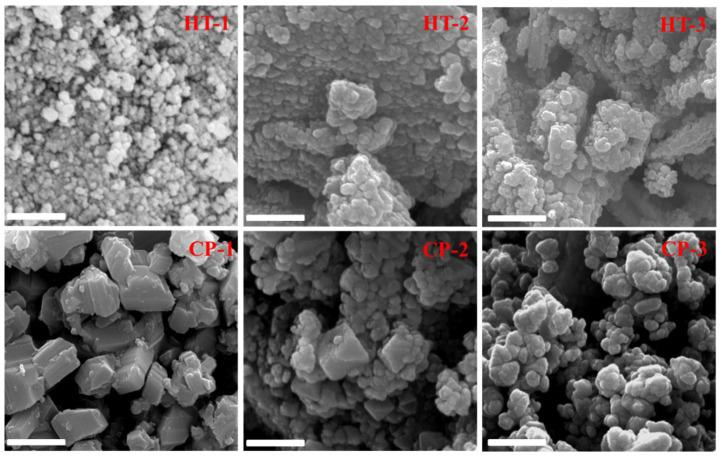
FE-SEM images of CeO_2_ NPs at different temperatures prepared via hydrothermal modification (HT) and wet chemical precipitation method (CP).

Additionally, as the morphology of the nanoparticles is a key characteristic factor for their performance, we investigated the effect of the preparation method with different temperatures on the CeO_2_ NPs morphology. Figure 4 presents typical FE-SEM images of CeO_2_ NPs prepared at different temperatures. As shown in the SEM images, the hydrothermally prepared CeO_2_ NPs exhibited spherical morphology with almost uniform characteristics. There is no obvious change in the morphology upon changing the temperature from 50 °C to 400 °C. The CeO_2_ NPs prepared by the wet chemical precipitation method showed a rough morphology compared with the hydrothermally modified analogous especially at a low temperature (50 °C). Upon increasing the temperature, the rough shape was regulated and semi-spherical NPs appeared as shown for CP-2 and CP-3. 

### 3.2. Effect of Solution pH

**Figure 5:**
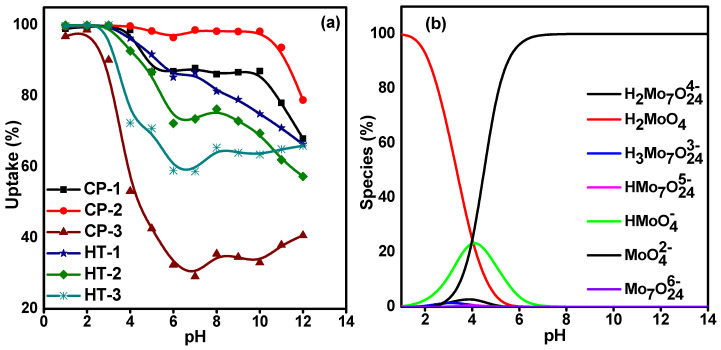
Effect of solution pH on (**a**) the Mo uptake on synthesized CeO_2_ NPs (C_o_ = 50 mg·L^−1^, V/m = 100 mL·g^−1^, and temperature = 25 ± 1 °C); (**b**) speciation of molybdenum.

Batch experiments were conducted to verify the usefulness of the synthesized CeO_2_ NPs for carrier-added ^99^Mo sorption from aqueous solutions. The pH of the Mo solution is the key factor that controls the sorption process, as it has a profound impact on determining the ionic state of the functional groups that exist on the sorbent surface. Furthermore, it affects the dissociation, complexation, and/or ionization of the adsorbed ions [30]. In order to predict the optimal experimental conditions for carrier-added ^99^Mo uptake, the sorption behavior of each material was evaluated at a wide range of pH values (1–12). The results are displayed in Figure 5a. The figure shows that the highest uptake values occur in an acidic medium (pH 2–4). After that, the uptake percent starts to decrease. This behavior can be explained based on two factors: the surface charge of the solid phase and the distribution of the molybdate species in the solution. 

Since the external solution pH governs the sorbents’ surface acid-base properties, we attempted to clarify the isoelectric point (ζ) of each material. Table 1 shows that the pH_IEP_ is reached at ~pH 7. Consequently, the CeO_2_ surface carries a positive charge at low pH (pH < 7). The change in the surface charge of the solid phase is because the active sites on the surface are hydrated and covered by amphoteric hydroxyl groups. Hence, based on the pH of the medium, these hydroxyl groups can undergo different reactions with either H^+^ or OH^−^ to develop positive or negative charges on the surface. Herein, at pH < 7, they are protonated, and the CeO_2_ surface develops a positive charge as follows:

(7) $$\mathrm{Surface}-{\mathrm{OH}}_{\mathrm{Surface}}+{\;H}_{\mathrm{solution}}^{+}\;⇌\;\mathrm{Surface}-{\mathrm{OH}}_{2}^{+}$$

Furthermore, the data in Figure 5a can be interpreted by considering the speciation diagram of molybdenum shown in Figure 5b. The data were calculated with the help of the PHREEQC software (version 3). Molybdenum exists in the solution as molybdate (MoO_4_^2−^). It polymerizes in an acidic medium to polymolybdate (Mo_7_O_24_^6−^) with higher molybdenum content per unit charge [31]. Therefore, an electrostatic attraction between negatively charged molybdate anions and positively charged surfaces occurs.

### 3.3. Effect of Contact Time

The progress of Mo sorption onto the synthesized CeO_2_ was studied at different time intervals, and the results are represented in Figure S1. The data illustrate that for all sorbents, the uptake percent (U%) of Mo increases with increasing the contact time until a sort of equilibrium state is reached, after which negligible or no further uptake takes place. In more detail, the sorption process took place in two distinct steps. The first one involves a fast uptake rate, which may be attributed to the high availability of active sites on the sorbent surface at the beginning of the sorption reaction. The second one exhibits a relatively slow sorption rate until the equilibrium is established. That is because the number of active sites is rapidly diminished, and the ions have to compete for the sorption sites [32]. Accordingly, under our experimental conditions, the optimum contact times for CP-1, CP-2, CP-3, HT-1, HT-2, and HT-3 are 6 h, 1 h, 30 h, 15 min, 1 h, and 10 h, respectively.

Furthermore, the results revealed that compared to materials prepared via the wet chemical method, the hydrothermally modified samples introduce a faster reaction rate. However, heated materials at 400 °C (CP-3 and HT-3) exhibit a relatively slow sorption rate. This behavior may be explained because the thermal treatment of nanomaterials decreases the number of active surface sites, which reduces the sorbent reactivity [32].

Based on the earlier experimental and surface morphological evaluations, the hydrothermal modification improved the sorption profile of HT-1 considerably. Moreover, except for CP-2, the thermal treatment does not enhance the uptake capability of the heated materials. On the contrary, it adversely diminishes the sorption rate for the ones heated at 400 °C. Therefore, CP-2 and HT-1 were selected for subsequent evaluation studies. 

#### 3.3.1. Kinetic Studies

To design an effective sorption process, determining kinetic parameters is crucial [33]. Kinetic modeling provides helpful information about the rate of the sorption process. Therefore, we analyzed the sorption kinetic data of carrier-added ^99^Mo onto CP-2 and HT-1 by using three different kinetic models, namely, Lagergren-first-order (Equation (8)) [34], pseudo-second-order (Equation (9)) [35], and Elovich (Equation (10)) [36,37]. These models are presented in a non-linear regression form by using the equations as follows:

(8) $$Q_{t}={\;Q}_{e}\left(1-\exp^{-K_{1}t}\;\right)\;$$

(9) $$Q_{t}=\frac{K_{2}Q_{e}^{2}t}{1+K_{2}Q_{e}t\;}$$

(10) $$Q_{t}=\frac{1}{\beta }\ln\left(1+\alpha \beta t\right)$$

where $Q_{t}\;$ and $Q_{e}$ (mg·g^−1^) are the amount of adsorbed molybdenum at a time (t) and equilibrium, respectively, $k_{1}$ (min^−1^) is the Lagergren-rate constant, ${\;k}_{2}$ (g/mg·min) is the pseudo-second-order rate constant, α (mg/g·min) is the Elovich rate constant, and β (mg·g^−1^) is the desorption constant representing the degree of surface coverage.

**Figure 6:**
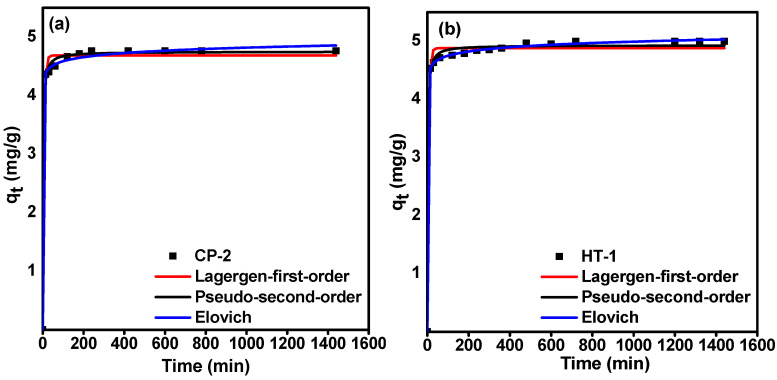
Non-linear fitting of kinetics models (Lagergren-first-order, pseudo-second-order, and Elovich) for the sorption of ^99^Mo on (**a**) CP-2; (**b**) HT-1.

Figure 6 displays non-linear fits of sorption kinetic data of Mo onto CP-2 and HT-1 according to the three aforementioned kinetic models by plotting calculated (q_t_) values versus contact time (t). The calculated kinetic parameters are presented in Table 2. Good non-linear plots are shown in Figure 6 for all kinetic models under our experimental conditions. Furthermore, by comparing the correlation coefficient values (R^2^) of the three applied models (Table 2), it can be seen that the three applied models have close R^2^ values. Nonetheless, we can notice that the Elovich model exhibits higher values (R^2^ = 0.999) than Lagergren-first-order model (R^2^ = 0.989 and 0.990) and the pseudo-second-order model (R^2^ = 0.991 and 0.990) for CP-2 and HT-1, respectively. Accordingly, we suggest that the Elovich model is the most appropriate to explain our sorption kinetics data. This finding may indicate that the prepared CeO_2_ matrices possess heterogeneous surfaces, and the uptake of carrier-added ^99^Mo develops multi-adsorption layers [36,37].

#### 3.3.2. Diffusion Studies

Generally, the uptake of adsorbate ions on the sorbent material occurs through [38]: (i) the movement of adsorbate ions from the bulk solution to the sorbent surface (bulk diffusion), (ii) the diffusion of adsorbate ions through the liquid film covering the sorbent surface (film diffusion), (iii) internal diffusion of adsorbate ions within the interior pores of the sorbent material (pore diffusion), and (iv) the binding of adsorbate ions on available active sites. In order to investigate the rate-limiting step, three diffusion models are applied. These models are film diffusion (McKay, Equation (11)) [39], intra-particle diffusion (Weber and Morris, Equation (12)) [40], and pore diffusion model (Bangham, Equation (13)) [40]. The models are expressed by the following equations:

(11) $$\;\ln\left(\frac{1-Q_{t}}{Q_{e}}\right)=-{\;k}_{f}t\;$$

(12) $$Q_{t}=K_{\mathrm{ip}}t^{\frac{1}{2}}+C\;$$

(13) $$\;\log\left(\log\left(\frac{C_{i}}{C_{i}-{\;Q}_{t}w}\;\right)\right)=\log\left(\frac{K_{b}W}{2.303\;V}\;\right)+\;\sigma \;\log\;t\;$$

where $Q_{t\;}$ and $Q_{e}$ (mg·g^−1^) are the amount of adsorbed molybdenum at a time (t) and equilibrium, respectively. C_i_ (mg·L^−1^) is the initial Mo concentration, W (g) is the sorbent mass, and V (L) is the solution volume. $k_{f}$ (min^−1^), $k_{\mathrm{ip}}$ (mg/g·min^1/2^), and σ are film diffusion, intra-particle diffusion, and pore diffusion rate constants, respectively. 

**Figure 7:**
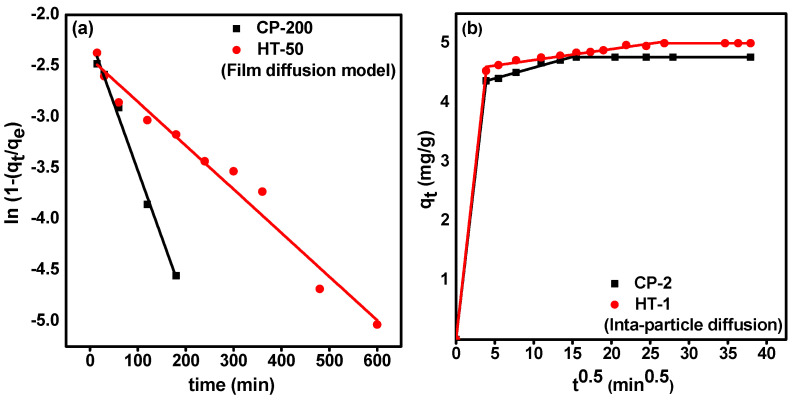

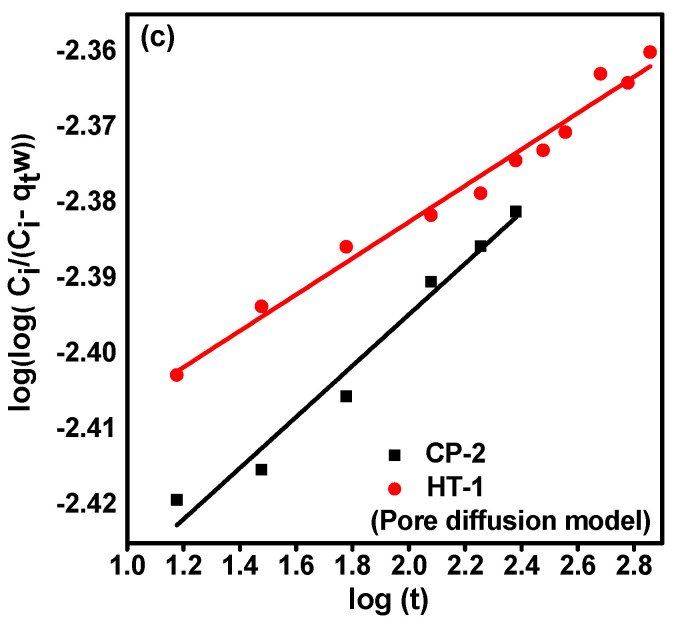
Diffusion plots of (**a**) McKay; (**b**) Weber and Morris; (**c**) Bangham for the sorption of carrier-added ^99^Mo on CP-2 and HT-1.

The rate constants can be determined from the slope of the linear plots of $\ln\left(\frac{1-q_{t}}{q_{e}}\right)$ versus t (Equation (11)), $q_{t}$ versus t (Equation (12)), and $\log\left(\log\left(\frac{C_{i}}{C_{i}-{\;Q}_{t}w}\;\right)\right)$ versus t (Equation (13)). The results are shown in Figure 7. The calculated diffusion parameters and the correlation coefficient values (R^2^) are tabulated in Table 3. In order to estimate the best mechanism that describes our diffusion data, we compare the correlation coefficient values (R^2^) of each model. For CP-2, the application of the McKay model resulted in a higher value (R^2^ = 0.992) than both Weber and Morris model (R^2^ = 0.977) and the Bangham model (R^2^ = 0.967). These findings indicate that the sorption of Mo on CP-2 might occur through an external film diffusion mechanism. For HT-1, the correlation coefficient values of the three models were: McKay model (R^2^ = 0.968), Weber and Morris model (R^2^ = 0.946), and Bangham model (R^2^ = 0.980). Therefore, it can be concluded that the pore diffusion model might control Mo sorption on HT-1.

### 3.4. Equilibrium Isotherm Studies

**Figure 8:**
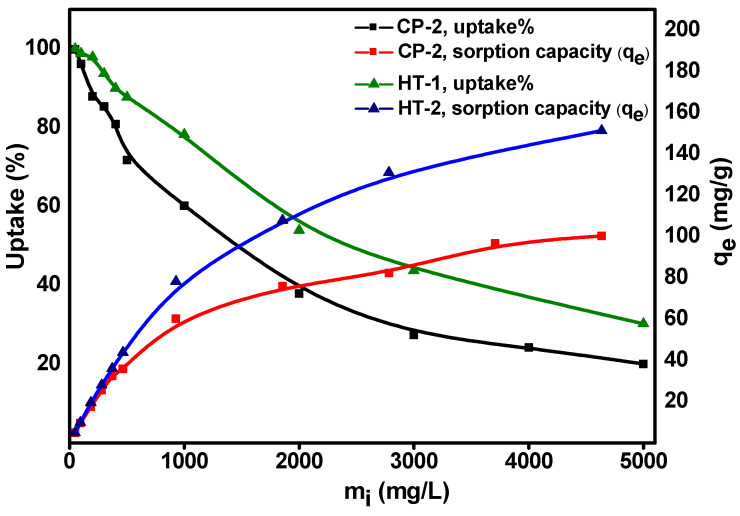
Effect of initial molybdate concentration on the uptake percent and equilibrium sorption capacity (Q_e_) of carrier-added ^99^Mo on CP-2 and HT-1 (pH = 3, V/m = 100 mL·g^−1^, t = 24 h, and temperature = 25 ± 1 °C).

Sorption isotherms describe how the adsorbed ions are dispersed between liquid and solid phases when the adsorption process reaches an equilibrium state at a constant temperature [41,42]. Consequently, a proper interpretation of equilibrium isotherms is necessary to improve the sorption mechanism and design an effective sorption system [31,43,44]. Figure 8 illustrates the influence of initial molybdate concentration on the equilibrium sorption capacity (Q_e_) and the uptake percent of Mo onto CP-2 and HT-1 at constant temperature (25 ± 1 °C). The figure shows that on increasing the initial Mo concentration, the uptake percent decreases, and on the contrary, the sorption capacity (Q_e_) increases. These findings are attributed to the increased mass driving force at higher molybdate concentrations [45,46,47].

Based on the sorbents’ behavior and the mode of interaction, the equilibrium isotherm data shown in Figure 8 were modeled. We applied three widely-used two-parameters, and isotherm models. These models are Freundlich [48], Langmuir [49], and Temkin [50]. The non-linear regressions of these models are represented by Equations (14)–(16), respectively:

(14) $$Q_{e}=K_{F}C_{e}^{\frac{1}{n}}$$

(15) $$Q_{e}=\frac{Q_{m}K_{l}C_{e}}{1+(K_{l}C_{e})}$$

(16) $$Q_{e}=\frac{\mathrm{RT}}{b_{t}}\ln\left(A_{t}C_{e}\right)$$

where Q_e_ (mg·g^−1^) is the equilibrium sorption capacity, C_e_ (mg·L^−1^) is the equilibrium concentration of molybdate ions, Q_m_ (mg·g^−1^) is the maximum capacity. K_F_ (mg^1-n^·L^n^/g), K_L_ (L·mg^−1^), and ${\;A}_{t\;}$ (L·g^−1^) are the Freundlich, Langmuir, and Temkin constants, respectively. The parameters *n* and *B* reflect the heterogeneity of adsorption sites and the heat of adsorption, respectively. R  is the universal gas constant (0.008314 kJ·mol^−1^·K^−1^), and T (K) is the absolute temperature. 

**Figure 9:**
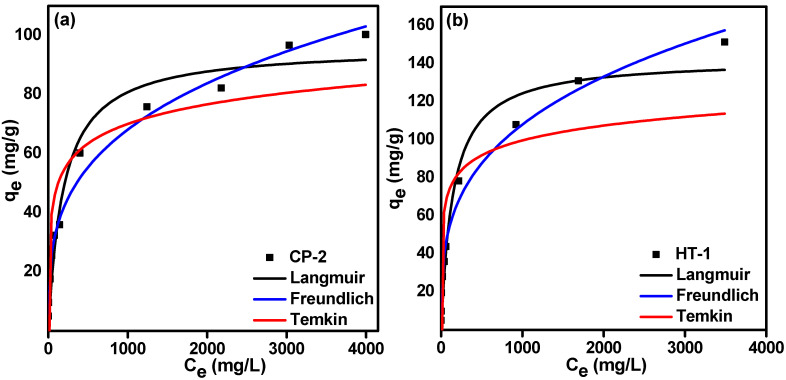
Equilibrium isotherms (Langmuir, Freundlich, and Temkin) of carrier-added ^99^Mo on (**a**) CP-2; (**b**) HT-1.

Figure 9 introduces the non-linear fits of isotherm data of Mo onto CP-2 and HT-1 for the three isotherms models mentioned above by plotting calculated (Q_e_) values versus (C_e_). The calculated equilibrium isotherm parameters and their corresponding correlation coefficient (R^2^) are given in Table 4. According to the correlation coefficient (R^2^) values, the Freundlich isotherm best describes the equilibrium data. It has a higher correlation coefficient (R^2^ = 0.986, 0.989) than Langmuir (R^2^ = 0.958, 0.983) and Temkin (R^2^ = 0.794, 0.756) for CP-2 and HT-1, respectively. This result is confirmed by the lower values of the error functions (X^2^), (APE), and (RMSE) for the Freundlich isotherm compared to the Langmuir and Temkin models (Table 4). Furthermore, the sorption intensity (n) values (for the Freundlich isotherm) are 3.35 (for CP-2) and 3.98 (for HT-1), which indicates a favorable sorption process. This result shows that CP-2 and HT-1 have heterogeneous surfaces with a wide distribution of non-equivalent sorption sites.

Furthermore, the maximum sorption capacity for Mo was evaluated under static conditions. We conducted repeated equilibrations of carrier-added ^99^Mo with CP-2 and HT-1 at 25 ± 1 °C until no further uptake occurred. The maximum sorption capacity was determined according to the following equation:

(17) $$Q_{\max}=\frac{\sum \mathrm{Uptake}\%}{100}\times {\;C}_{o}\times \frac{V}{m}$$

where $Q_{\max}$ (mg·g^−1^) is the maximum (saturation) sorption capacity, $C_{o}$ (mg·L^−1^) is the initial concentration,  V (L) is the volume of the aqueous phase, and m (g) is the matrix weight. 

The results show that the sorption capacities of CP-2 and HT-1 reach 184 ± 12 and 192 ± 10 mg Mo·g^−1^, respectively. These values might be attributed to the high surface area of CP-2 and HT-1. In addition, the sorption behavior of each sorbent allows the formation of multi-adsorption layers on their surfaces, which is confirmed by kinetic and equilibrium isotherm studies. It is pertinent to point out that the sorption capacities of CeO_2_ nanosorbents we developed are much higher than the sorption capacity of conventional alumina (2–20 mg Mo·g^−1^) [8]. Therefore, the new materials have the potential to be implemented to produce ^99^Mo/^99m^Tc generators by using LSA ^99^Mo.

### 3.5. Thermodynamic Studies

The effect of temperature on molybdenum uptake onto CP-2 and HT-1 was studied in the temperature range (298–343 K). With increasing the reaction temperature, a slight increase in carrier-added ^99^Mo uptake is observed on HT-1. Meanwhile, the sorption capacity of CP-2 increases noticeably. These findings indicate that the sorption process is endothermic in nature.

In order to understand the thermodynamic behavior of the sorption process, we investigated the values of the enthalpy change (ΔH°), the entropy change (ΔS°), and the free energy change (ΔG°) in our experimental temperature window. These parameters were calculated by using Vant’ Hoff equations [51], as follows:

(18) $${\mathrm{lnK}}_{d}=\left(-\frac{\Delta H^{{}^{\circ}}}{\mathrm{RT}}\right)+\left(\frac{\Delta S^{{}^{\circ}}}{R}\right)$$

(19) $$\Delta G^{{}^{\circ}}=\Delta H^{{}^{\circ}}-T\Delta S^{{}^{\circ}}\;$$

(20) $$\Delta G^{{}^{\circ}}=-{\mathrm{RTlnK}}_{d}$$

where K_d_ (mg·mL^−1^) is the distribution coefficient, R is the universal gas constant (0.008314 kJ·mol^−1^·K^−1^), and T (K) is the absolute temperature. 

**Figure 10:**
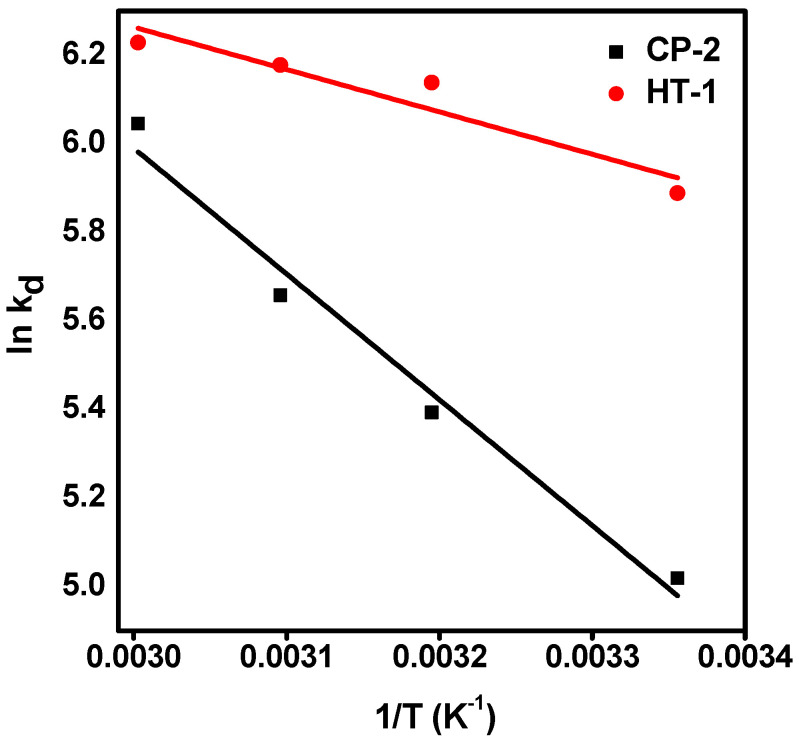
Van’t Hoff plot for the sorption of carrier-added ^99^Mo on CP-2 and HT-1 (C_o_ = 1000 mg·L^−1^, pH = 3, V/m = 100 mL·g^−1^, and t = 24 h).

Figure 10 shows linear plots of lnK_d_ versus the reciprocal absolute temperature (1/T). The values of ∆H° and ∆S° can be determined from the slope and intercept, respectively. The calculated values of ΔH°, ΔS°, and ∆G° are listed in Table 5. The positive values of ΔH° indicate that the sorption processes of Mo onto CP-2 and HT-1 are endothermic. In addition, the positive values of ΔS° point to the increase in randomness at the solid–liquid interface. Moreover, the negative values of ∆G° confirm the spontaneous nature of the sorption processes under our experimental conditions [52]. 

## 4. Summary and Conclusions

Our main goal was to evaluate the sorption profile of LSA ^99^Mo on several CeO_2_ nanosorbents developed in our laboratory. First, these materials were synthesized by using simple wet-chemical precipitation (CP) and hydrothermal (HT) methods. Then, they were heated at different temperatures: 200 °C and 400 °C. Eventually, an assessment of the carrier-added ^99^Mo uptake behavior was carried out under different experimental conditions. We found that the hydrothermal modification improves the morphological and sorption profile of HT-1 considerably. Except for CP-2, the thermal treatment did not enhance the sorption capability of the heated materials. In contrast, it diminished the Mo sorption for the ones heated at 400 °C. In addition, sorption kinetics data of carrier-added ^99^Mo Mo onto CP-2 and HT-1 were most favorably described by the Elovich model. The diffusion data showed that film and pore diffusion models controlled the sorption process for CP-2 and HT-1, respectively. Moreover, out of the investigated isotherm models, the Freundlich model was the most appropriate to describe the equilibrium isotherms of carrier-added ^99^Mo on both sorbents. Furthermore, we studied the influence of reaction temperature on Mo uptake, and the results confirmed the spontaneous and endothermic nature of the sorption processes. Finally, we evaluated the maximum sorption capacity of CP-2 and HT-1 towards carrier-added ^99^Mo under static sorption conditions. Their capacity reached 184 ± 12 and 192 ± 10 mg Mo.g^–1^, respectively. From the outcome of our investigation, it is possible to conclude that HT-1 and CP-2 have favorable sorption profiles and appreciable uptake capacities for carrier-added ^99^Mo. Consequently, they have the potential for producing a ^99m^Tc radionuclide generator by using LSA ^99^Mo. Clearly, the next stage of our research will investigate the feasibility of using these two sorbent materials to develop a useful ^99^Mo/^99m^Tc generator.

## Figures and Tables

**Table 1:** BET surface area (S_BET_), average pore volume and diameter, crystallite size, and isoelectric point (pH_IEP_) for CeO_2_ NPs prepared at different conditions.

Sample	S_BET_, (m^2^·g^−^^1^)	Pore Volume, (cm^3^·g^−^^1^)	Pore Size, (nm)	Crystallite Size, (nm)	pH_IEP_
CP-1	150.2	0.085	2.26	2.50	6.91
CP-2	175.3	0.057	2.38	4.62	6.92
CP-3	97.4	0.031	2.33	5.66	6.94
HT-1	187.2	0.128	2.75	3.54	6.99
HT-2	179.8	0.122	2.71	3.61	7.00
HT-3	114.7	0.078	2.71	3.50	7.02

**Table 2:** Kinetic parameters for the sorption of carrier-added ^99^Mo on CP-2 and HT-1.

Kinetic Model	Parameter	CP-2	HT-1
Lageregen-first-order	q_e,1_ (mg·g^−1^)	4.686	4.881
	K_1_ (min^−1^)	0.171	0.170
	R^2^	0.990	0.989
Pseudo-second-order	q_e,2_ (mg·g^−1^)	4.751	4.930
	K_2_ (g/mg·min)	0.128	0.127
	R^2^	0.991	0.990
Elovich	α (mg/g·min)	$4.724\times 10^{16}$	3.877 × 10^16^
	β (g·mg^−1^)	9.874	9.48458
	R^2^	0.999	0.999

**Table 3:** Diffusion parameters for the sorption of carrier-added ^99^Mo on CP-2 and HT-1.

Diffusion Model	Parameter	CP-2	HT-1
McKay (Film diffusion)	${\;k}_{f}\;$(min^−1^)	0.013	0.004
	intercept	−2.218	−2.425
	R^2^	0.992	0.968
Weber and Morris (Intra-particle diffusion)	$k_{\mathrm{ip}\;}$(mg/g·min^1/2^)	0.036	0.019
	Intercept (C) *	4.224	4.531
	R^2^	0.977	0.946
Bangham (Pore diffusion)	σ **	0.034	0.024
	intercept	−2.462	−2.431
	R^2^	0.967	0.980

* The intercept is influenced by the boundary layer thickness. ** σ value should be <1.

**Table 4:** Isotherm parameters and error functions calculations for the sorption of carrier-added ^99^Mo on CP-2 and HT-1.

Isotherm Model	Parameter	CP-2	HT-1
Langmuir	Q_max_ (mg·g^−^^1^)	96.083	109.114
	K_L_ (L·mg^−^^1^)	0.005	0.012
	R^2^	0.958	0.983
	X^2^	41.989	138.843
	APE	28.75	36.032
	RMSE	7.190	11.185
Freundlich	K_F_ (mg^1-n^·L^n^·g^−^^1^)	8.668	15.078
	n	3.350	3.981
	R^2^	0.986	0.989
	X^2^	3.888	4.678
	APE	11.442	15.304
	RMSE	4.060	5.524
Temkin	b_t_	0.260	0.266
	A_t_ (L·g^−^^1^) *	1.535	8.199
	R^2^	0.794	0.756
	X^2^	32.407	70.355
	APE	81.781	137.723
	RMSE	15.917	28.175

* A_t_ is the equilibrium-binding constant that corresponds to the maximum binding energy.

**Table 5:** Thermodynamic parameters for the sorption of carrier-added ^99^Mo on CP-2 and HT-1.

CeO_2_ NPs	Temperature (K)	ΔG° (kJ·mol^−1^)	ΔH° (kJ·mol^−1^)	ΔS° (kJ·mol^−1^·K^−1^)	R^2^
CP-2	298	−12.338	23.620	0.121	0.970
	313	−14.149			
	323	−15.355			
	333	−16.562			
HT-1	298	−14.676	7.959	0.076	0.867
	313	−15.816			
	323	−16.575			
	333	−17.335			

## Data Availability

Data is contained within the article.

## Supplementary Materials

The following supporting information can be downloaded at: https://www.mdpi.com/article/10.3390/nano12091587/s1, Figure S1: Effect of contact time on Mo uptake on synthesized CeO_2_ NPs.
