# Supplementary material for: Sorption Profile of Low Specific Activity 99Mo on Nanoceria-Based Sorbents for the Development of 99mTc Generators: Kinetics, Equilibrium, and Thermodynamic Studies
Source: Nanomaterials (Basel). 2022 May 7;12(9):1587. doi: 10.3390/nano12091587 (PMC9102646; doi:10.3390/nano12091587)
Supplement: Supplementary file 1 [file nanomaterials-12-01587-s001.zip › nanomaterials-1694882-supplementary.pdf]

Supplementary Information

Sorption Profile of Low Specific Activity ^{99}Mo on Nanoceria-Based Sorbents for the development of ^{99m}Tc Generators: Kinetics, Equilibrium, and Thermodynamic Studies

Mohamed F. Nawar [1*], Alaa F. El-Daoushy [2], Metwally Madkour [3], and A. Türler [1]

1 Department of Chemistry, Biochemistry, and Pharmaceutical Sciences, Faculty of Science, University of Bern, Freiestrasse 3, CH-3012 Bern, Switzerland.

2 Radioactive Isotopes and Generators Dept., Hot Laboratories Center, Egyptian Atomic Energy Authority, 13759, Cairo, Egypt

3 Chemistry Department, Faculty of Science, Kuwait University, 5969, Safat, 13060, Kuwait.

* Correspondence: mohamed.nawar@unibe.ch; mohamedfnawar@yahoo.com

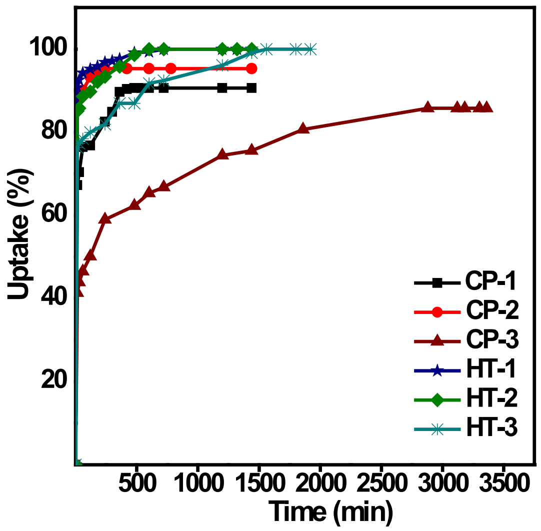

Figure S1. Effect of contact time on Mo uptake on synthesized CeO_2 NPs (C_o = 50 mg.L^{-1}, pH = 3, V/m = 100 mL.g^{-1}, and temp = 25 ± 1 ^{o}C).
